# Fasting Glucagon Level in Type 2 Diabetes and Impaired Glucose Tolerance and Its Association With Diabetes-Associated Clinical Parameters: A Study From Karachi, Pakistan

**DOI:** 10.7759/cureus.13430

**Published:** 2021-02-18

**Authors:** Eraj Abbas, Iftikhar Ahmed Siddiqui, Muhammad Saeed Khan, Kahkashan Perveen, Anum Butt, Asher Fawwad

**Affiliations:** 1 Biochemistry, Baqai Medical University, Karachi, PAK; 2 Research, Baqai Institute of Diabetology and Endocrinology, Baqai Medical University, Karachi, PAK

**Keywords:** glucagon, type 2 diabetes, impaired glucose tolerance, obesity

## Abstract

Aim and objective

The study aims to analyze fasting glucagon in patients with type 2 diabetes and impaired glucose tolerance and correlate it with anthropometric and biochemical parameters in a large proportion of Pakistani people with diabetes.

Methodology

The participants of the study were categorized into three groups based on oral glucose tolerance test, as per American Diabetes Association guidelines. Group A consisted of normal glucose tolerance subjects (n=30), Group B consisted of subjects with impaired glucose tolerance (n=30), and Group C had full-blown subjects with type 2 diabetes (n=30). Biochemical parameters, such as fasting glucagon, fasting plasma and 2-hour glucose, glycated hemoglobin, and lipid profile, and anthropometric parameters, such as body mass index (BMI), waist and hip circumference, waist-to-hip ratio, and systolic and diastolic blood pressure, were measured.

Results

The mean values of fasting glucagon level in Group A, Group B, and Group C were 39.24±4.5, 44.5±8.25, and 49.02±9.15 pg/ml, respectively. Statistically significant difference was not found in fasting glucagon level among these groups (p-value 0.614). Fasting glucagon was positively and independently correlated with 2-hour plasma glucose, systolic blood pressure, diastolic blood pressure, BMI, hip and waist circumference, and hip-to-waist ratio in Group C. In Group B, fasting glucagon was positively correlated with 2-hour plasma glucose, BMI, and hip circumference, while it was not correlated with fasting plasma glucose in both groups. In Group A, fasting glucagon found positively correlated with systolic blood pressure and hip circumference.

Conclusion

Our observation suggests that fasting plasma glucose is not concomitant with glucagon levels; however, glucagon suppression, after glucose intake, was dysregulated in type 2 diabetes and impaired glucose tolerance. Moreover, glucagon is associated with central obesity in type 2 diabetic patients.

.

## Introduction

The primary cause of type 2 diabetes mellitus (T2DM) is insulin resistance and relative insulin deficiency; however, many subjects also exhibit inappropriate levels of circulating glucagon [1]. Important evidence for this was the demonstration that subjects with T2DM had higher baseline glucagon levels than subjects without diabetes, and these levels were not suppressed by a carbohydrate meal, as was the case in subjects without diabetes [2].

Glucagon elevates the concentration of glucose in the blood upon fasting by activating gluconeogenesis (i.e., the hepatic production of glucose through a non-carbohydrate source) and by the breakdown of glycogen [3]. Regulation of glucagon secretion is impaired in subjects with T2DM who have elevated plasma glucagon concentrations in the fasting state and defective postprandial glucagon suppression that results in undesirably high plasma glucagon concentrations in the context of hyperglycemia [4]. Lack of suppression of postprandial glucagon secretion in subjects with T2DM plays an important role in the pathogenesis of postprandial hyperglycemia [5].

Thus, hyperglucagonemia contributes to the increased hepatic glucose production that distinguishes subjects with T2DM. Furthermore, in subjects with T2DM, plasma glucagon concentrations may even increase in response to a meal [6]. The mechanisms accountable for increased glucagon levels under hyperglycemic conditions in subjects with T2DM may include impaired glucose sensing by alpha cells and/or resistance of pancreatic alpha cells to the inhibitory actions of insulin [7].

Obesity is associated with insulin resistance and increases the risk of T2DM development. An excess of adipose tissue induces insulin resistance and pancreatic beta-cell dysfunction modulated by releasing non-esterified fatty acids; endocrine hormones, such as leptin and adipokine; and proinflammatory cytokines [8]. Stern et al. [9] recently reported that the dysregulation of glucagon secretion in obesity leads to glucoregulatory disturbance in mice. In contrast, glucagon appears to promote satiety and increased energy expenditure in obese individuals, which may contribute to weight loss. Also, insulin and glucagon have a role in energy balance regulation [10,11]. The current study is, therefore, designed to analyze serum glucagon levels in subjects with T2DM and impaired glucose tolerance compared to normal glucose tolerance and to evaluate the correlation of fasting glucagon with fasting and 2-hour plasma glucose (2-hPG), glycated hemoglobin (HbA1c), lipid profile, and anthropometric parameters specifically with the parameters of central obesity in the study population.

## Materials and methods

The study was conducted at Baqai Institute of Diabetology and Endocrinology, Baqai Medical University (BMU), Karachi for the duration of January 2016 to January 2018. Convenience sampling for the research purpose was used from the available pool of respondents. The participants of this study were categorized into three groups on the basis of oral glucose tolerance test (OGTT), as per American Diabetes Association (ADA) guidelines. Group A consisted of normal glucose tolerance subjects (n=30), Group B consisted of subjects with impaired glucose tolerance (n=30), and Group C had full-blown subjects with T2DM (n=30). Normal glucose tolerance was taken as fasting plasma glucose level (FPG) <100 mg/dl and 2-hPG level <140 mg/dl. Impaired glucose tolerance was considered as FPG level 100-125 mg/dl and 2-hPG level 140-199 mg/dl. Diabetes was considered as FPG level ≥126 mg/dl and 2-hPG level ≥200 mg/dl as per ADA guidelines [12]. Subjects with known comorbidities such as cancer or other debilitating disorders such as cardiopulmonary disorders, an autoimmune disorder, type 1 diabetes, and gestational diabetes were excluded. The study was carried out after ethical approval from the ethics committee of BMU.

Anthropometric measures

Weight, height, waist and hip circumference, and blood pressure were measured using standardized methods [13]. Body mass index (BMI) was calculated as weight in kilograms divided by height in meters squared (kg/m^2^). Waist circumference was measured between the center point of the lower margin of the ribs and the mid-point of the iliac crest. Hip circumference was measured around the widest portion of the buttocks, with the tape parallel to the floor [14]. Blood pressure was measured using an auscultatory method with the help of a stethoscope and a sphygmomanometer [15].

Clinical measures

Biochemical parameters such as fasting and 2-hPG, HbA1c, lipid profile, and glucagon were assessed by standardized methods. Fasting glucagon was estimated by the glucagon enzyme immunoassay using human glucagon enzyme-linked immunosorbent assay kit (Genorise Scientific, Inc., PA, USA) [2]. Fasting plasma glucose and 2-hPG levels were measured using glucose oxidase peroxidase [16]; total cholesterol was estimated using the cholesterol oxidase phenol 4-amino antipyrine peroxidase method, and triglycerides were measured using the glycerol phosphate oxidase-p-aminophenazone method. High-density lipoprotein cholesterol (HDL-C) was estimated using the Immuno FS method, low-density lipoprotein cholesterol (LDL-C) was measured by the direct method [17], and HbA1c was measured by high-performance liquid chromatography [18].

Statistical analysis

All statistical analyses were performed using IBM SPSS Statistics for Windows, Version 20.0. (IBM Corp., Armonk, NY, USA). The anthropometric and clinical variables were analyzed by using either the one-way analysis of variance or Student’s t-test, Mann-Whitney U test or Kruskal-Wallis H test, and chi-square test as appropriate. The degree of correlation between metabolic parameters and glucagon was presented by the Pearson correlation coefficient. Multiple linear regression analyses assessed the independent predictors of glucagon. Results were considered statistically significant at a p-value <0.05.

## Results

The mean of anthropometric and biochemical parameters among study groups is compared in Table 1. It is shown that mean age, weight, hip and waist circumference, and systolic blood pressure were significantly higher in Group C subjects as compared to Group A, whereas no significant difference was found in any parameter between Group B and Group C. The mean difference of fasting glucagon was not found significant among the study group. Fasting plasma glucose and 2-hPG levels were significantly higher in Group C and Group B (p-value < 0.0001) as compared to Group A. Cholesterol, triglycerides, and LDL level were significantly higher in Group C as compared to Group A, while HDL level was lower, respectively. HbA1c was found significantly higher in Group C as compared to the other two groups.

**Table 1:** Comparison of the baseline anthropometric and biochemical characteristics between Group A, Group B, and Group C p-Value ≤0.05 is taken as significant; *p ≤ 0.05 (Group A vs. Group B); **p ≤ 0.05 (Group A vs. Group C); *p ≤ 0.05; †p ≤ 0.05 (Group B vs. Group C). BMI, body mass index; SBP, systolic blood pressure; DBP, diastolic blood pressure; HDL, high-density lipoprotein; LDL, low-density lipoprotein; HbA1c, glycated hemoglobin. Group A: Patients with normal glucose tolerance. Group B: Patients with impaired glucose tolerance. Group C: Patients with type 2 diabetes.

Parameters	Group A	Group B	Group C
Age (years)	44.17 ± 12.86	45.79 ± 12.6	52.03 ± 9.88**
BMI (kg/m^2^)	26.21 ± 4.53	28 ± 5.51	27.8 ± 6.4
Waist circumference (cm)	91.48 ± 12.79	92.7 ± 11.84	98.67 ± 14.35**
Hip circumference (cm)	67.76 ± 14.42	102.73 ± 18.21	105.63 ± 12.9**
SBP (mmHg)	120.9 ± 17.02	125.17 ± 17.65	131.87 ± 23.01**
DBP (mmHg)	81.17 ± 10.69	81.03 ± 9.39	85.53 ± 10.89
Fasting glucagon (pg/ml)	39.24 ± 4.5	44.51 ± 8.25	49.02 ± 9.15
Fasting plasma glucose (mg/dl)	87.83 ± 10.44	107.73 ± 10.85*	202.97 ± 10.23**
Two-hour plasma glucose (mg/dl)	100.93 ± 21.46	145.97 ± 16.89*	275.64 ± 13.32**
Cholesterol (mg/dl)	220.43 ± 14.17	236.43 ± 12.31	248.9 ± 9.74**
Triglyceride (mg/dl)	163.63 ± 9.21	203.1 ± 8.49*	216.37 ±14.21**
HDL (mg/dl)	38.43 ± 10.49	36.97 ± 5.93	20.47 ± 6.28**
LDL (mg/dl)	115.53 ± 12.51	108.83 ± 6.19	111.07 ± 12.31**
HbA1c (mg/dl)	5.35 ± 0.56	5.52 ± 0.7†	6.83 ± 1.82**

The statistics of correlation were applied to investigate the effect of fasting glucagon on anthropometrics and biochemical parameters in study groups. The incidence of positive correlation was found for fasting glucagon with 2-hPG, systolic blood pressure, diastolic blood pressure, BMI, waist circumference, and waist-to-hip ratio in Group C (p < 0.05). The fasting glucagon was positively correlated with 2-hPG and BMI in Group B and with systolic blood pressure in Group A. However, fasting glucagon was positively associated with hip circumference among all groups (p < 0.05) (Table 2).

**Table 2:** Correlation of fasting glucagon with anthropometric and biochemical characteristics of Group A, Group B, and Group C *p-Value ≤0.05 is taken as significant. BMI, body mass index; SBP, systolic blood pressure; DBP, diastolic blood pressure; HDL, high-density lipoprotein; LDL, low-density lipoprotein; HbA1c, glycated hemoglobin Group A: Patients with normal glucose tolerance. Group B: Patients with impaired glucose tolerance. Group C: Patients with type 2 diabetes.

Parameters	Group A	p-Value	Group B	p-Value	Group C	p-Value
BMI	0.090	0.636	0.383	0.051*	0.387	0.05*
Age	-0.270	0.183	-0.270	0.183	-0.356	0.135
Waist circumference	0.011	0.952	0.307	0.127	0.370	0.047*
Hip circumference	0.421	0.021*	0.483	0.012*	0.521	0.022*
Waist-to-hip ratio	0.028	0.876	0.206	0.221	0.472	0.031*
SBP	0.391	0.033*	0.037	0.858	0.441	0.051*
DBP	0.141	0.456	0.160	0.436	0.446	0.052*
Fasting plasma glucose	0.224	0.119	0.204	0.318	0.398	0.091
Two-hour plasma glucose	0.101	0.546	0.421	0.021*	0.470	0.041*
HbA1c	0.199	0.118	-0.145	0.457	-0.216	0.117
Cholesterol	-0.167	0.378	0.202	0.321	0.133	0.586
Triglyceride	0.011	0.956	-0.179	0.381	0.158	0.518
HDL	0.146	0.441	0.146	0.457	0.286	0.235
LDL	0.042	0.824	0.199	0.329	0.222	0.361

Multiple linear regression analysis derived 2-hPG, SBP, DBP, BMI, waist and hip circumference, and waist-to-hip ratio as the independent predictors of fasting glucagon in Group C (p < 0.05). In Group B, 2-hPG, BMI, and hip circumference were evaluated as independent predictors of fasting glucagon, while SBP and hip circumference were found independently and positively associated with fasting glucagon in Group A (p < 0.05) (Table 3).

**Table 3:** Multiple linear regression analysis expressing independent predictors of fasting glucagon in Group A, Group B, and Group C *p-Value ≤0.05 is taken as significant. BMI, body mass index; SBP, systolic blood pressure; DBP, diastolic blood pressure Group A: Patients with normal glucose tolerance. Group B: Patients with impaired glucose tolerance. Group C: Patients with type 2 diabetes.

Studied groups	Clinical variables	Standardized coefficients (β)	p-Value
Group A	SBP	0.391	0.033*
	Hip circumference	0.421	0.021*
Group B	Two-hour plasma glucose	0.421	0.021*
	BMI	0.383	0.051*
	Hip circumference	0.483	0.012*
Group C	Two-hour plasma glucose	0.470	0.041*
	SBP	0.441	0.051*
	DBP	0.446	0.052*
	BMI	0.387	0.05*
	Waist circumference	0.370	0.047*
	Hip circumference	0.521	0.022*
	Waist-to-hip ratio	0.472	0.031*

## Discussion

In the current study, we observed fasting glucagon level in patients with T2DM, impaired and normal glucose tolerance, and found insignificant differences in fasting glucagon level among study groups. This finding is consistent with the previous study indicating that glucose tolerance may not significantly affect fasting glucagon level [19]. However, conflicting finding has been reported, showing significantly higher fasting glucagon level in type 2 diabetic subjects [20]. A positive correlation of fasting glucagon with 2-hour postprandial plasma glucose levels in Groups B and C was found, thereby indicating dysregulated glucagon suppression after glucose intake. This finding is in line with the previous research that was done by Abdul-Ghani and DeFronzo [21]. Patients with T2DM have impaired postprandial glucagon suppression in the circumstances of defective insulin secretion and/or action [22]. However, it is of revelation in the present study that fasting glucagon was not correlated with FPG level and HbA1c level among any of the study groups. It is controversial as to whether there is a significant association between glucagon levels and glycemic control, as Knop et al. and Yoshiya et al. also validated our findings and suggested that fasting glucagon was not correlated with the FPG concentration in people with diabetes and impaired glucose tolerance, and, therefore, similar in diabetic and non-diabetic subjects [23,24]. Ortega et al. [25] reported that circulating glucagon levels were positively correlated with fasting glucose and HbA1C levels in obese subjects with impaired glucose tolerance. In contrast, Abdul-Ghani and DeFronzo found that glucagon concentration was inversely correlated with the FPG concentrations (r = -0.35; P = 0.001) during the OGTT, in T2DM participants [21]. This may be due to the difference in the measurement methods of glucagon levels or due to the specific characteristics of the study population.

Glucagon is known to increase energy expenditure through thermogenesis in brown adipose tissue [26]. Cegla et al. [27] reported that glucagon infusion increased the respiratory exchange ratio, resting energy expenditure rate, and carbohydrate and fat oxidation rates in healthy individuals. A systematic review and meta-analysis revealed a parallel decrease between fasting glucagon levels and weight loss in obese subjects [28]. In contrast, the current study identified a strong positive association of fasting glucagon level with BMI, waist circumference, waist-to-hip ratio in Group C, and hip circumference among all study groups. Thereby, it is seen that the appropriate balance between insulin and glucagon that is deficient in diabetes has a more favorable impact on weight control. It is also evident from the previous report that glucagon levels were elevated in high-hip circumference subjects and independently associated with other measures of abdominal obesity [20]. We also found the same outcomes and observed that glucagon was independently and positively associated with measures of central obesity in the current study. The role of glucagon in the pathophysiology of diabetes has long been recognized [8]. There are many modified molecules of glucagon that had the property to inhibit the function of glucagon receptors, thus preventing the hyperglucagonemia-induced hyperglycemia seen in diabetes [29]; however, the association of glucagon with abdominal obesity has been sparsely researched, and only a few studies are available. The present study indicated a positive association of fasting glucagon level with central obesity in type 2 diabetic subjects.

Research shows that glucagon acts primarily on the liver through regulation of hepatic lipid metabolism with reduction of the hepatic lipid accumulation and decrease in hepatic lipid secretion. Regarding the whole-body lipid metabolism, it is controversial to what extent glucagon influences lipolysis in adipose tissue, particularly in humans [30]. Physiologically, in the fasting state, exogenous glucagon reduces triglycerides, cholesterol levels, and release of LDL via the glucagon receptor with stimulated fatty acid oxidation, leading to the release of free fatty acids [30]. In contrast, Shajith et al. observed a significantly positive correlation of glucagon levels with LDL levels [20]. In the current study, we found no significant correlation of fasting glucagon level with lipid profile in the study population. The cause and significance of this observation are not clear; however, the altered liporegulatory role of glucagon due to excess adiposity in the study population may be contributory. The study is not without limitation. The role of glucagon in obese diabetic subjects would be more enlightening if a large sample size was selected for the research.

Future recommendations

Future studies with a large number of participants and other measurement parameters may further reinforce our current findings and help to understand the pathophysiological role of glucagon in diabetes. The sample size is small, especially for lean participants. The association of glucagon with clinical and anthropometric parameters would more enlighten with a large sample size and equal disparity of lean and obese subjects. The evaluation of other clinical parameters associated with glucose hemostasis might be assessed like insulin, insulin resistance, C-peptide, and glucagon-to-insulin ratio.

## Conclusions

In summary, we report a positive association of glucagon level with 2-hour postprandial plasma glucose levels may indicate poor suppression of glucagon. Our findings also suggest that glucagon level is significantly associated with abdominal obesity in type 2 diabetic subjects; therefore, glucagon-suppressive drugs for the treatment of diabetes may have a significant effect on weight management. Additional studies are necessary to confirm and further clarify the clinical implications of fasting glucagon levels with glucose hemostasis and abdominal obesity.
